# Prevalence of cardiovascular disease risk factors in people of Asian Indian origin: Age and sex variation

**DOI:** 10.4103/0975-3583.64441

**Published:** 2010

**Authors:** Arnab Ghosh, Minakshi Bhagat, Mithun Das, Sanjib Kumar Bala, Riddhi Goswami, Susil Pal

**Affiliations:** *Biomedical Research Laboratory, Department of Anthropology, Visva Bharati University, Santiniketan, West Bengal, India*; 1 *Department of Anthropology, Sree Chaitanya College, Habra, West Bengal, India*; 2 *Department of Biotechnology, Heritage Institute of Technology, Kolkata, India*; 3 *Human Genetic Engineering Research Centre, Kolkata, India*

**Keywords:** Aging, Asian Indians, cardiovascular disease, metabolic syndrome, obesity

## Abstract

**Background:**

No study has been undertaken on people of Asian Indian origin to investigate the age and sex variation in the prevalence of cardiovascular disease (CVD) risk factors.

**Objectives:**

To investigate the age and sex variation in the prevalence of CVD risk factors among the people of Asian Indian origin.

**Materials and Methods:**

A total of 682 (302 males and 380 females) participants aged 25–85 years took part in the study. The subjects were categorized into 4 groups, namely, Group I (25–34 years), Group II (35–44 years), Group III (45–54 years), and Group IV (55 years and above). Height, weight, and the circumferences of minimum waist (MWC) and maximum hip were collected using standard techniques. Waist–hip ratio (WHR) was then calculated. Percentage of body fat (%BF) and body mass index (BMI) were measured using an Omron body fat analyzer. Left arm systolic (SBP) and diastolic (DBP) blood pressure were taken from each participant with the help of an Omron MI digital electronic blood/pulse monitor. Metabolic profiles, namely, total cholesterol (TC), triglyceride (TG), high (HDL), low (LDL), very low-density lipoprotein (VLDL), and fasting blood glucose (FBG) were also measured using an autoanalyzer.

**Results:**

One-way analysis of variance revealed significant differences for age, BMI, MWC, WHR, SBP, DBP, TC, TG, LDL, VLDL, and TC:HDL and TG:HDL ratios across the groups. It was observed that there were significant sex-specific group differences (male [χ^2^ (12)] =29.22, *P* < 0.01 and female [χ^2^ (12)] =56.69, *P* < 0.001) for obesity, high BP, high TC, high TG, and high FBG. But no significant group-specific sex difference was evident for either of the risk factors, except for Group IV.

**Conclusion:**

Age irrespective of sex modulates CVD risk factors and warranted prevention as early as middle age.

## INTRODUCTION

Cardiovascular disease (CVD) is now a days the leading cause of death for men and women both in the developed and developing countries.[1] It was reported that the mortality from CVD was projected to decline in the developed countries from 1970 to 2015, while it was projected to almost double in the developing countries.[2]

The prevalence of coronary heart disease (CHD) is known to be high in people of south Asian descent (subjects originally from Indian subcontinent). Some metabolic abnormalities are more prevalent among them, including high triglyceride (TG) concentration, increased total cholesterol (TC) and high-density lipoprotein (HDL) ratio, type 2 diabetes mellitus (T2DM), and central or visceral obesity.[3]

For men and women, cardiovascular risk is known to increase with age, smoking, hypertension, blood lipids and glucose levels, and central obesity.[4] Despite that over the lifespan, approximately the same proportion of the female population as the male population dies of complications resulting from CVD, it has been traditionally considered as a middle-aged "male" disease, the consequence has been for long, the exclusion of women from clinical trial and epidemiologic studies, making extensive to women the results obtained for men. In the 1970s, it was suggested that endogenous hormones protect against CVD in women, and that estrogen deprivation after menopause increased their cardiovascular risk.[5] It was also evident that among the Asian Indian women, postmenopausal women were more susceptible to diabetes and cardiovascular diseases than premenopausal women.[6]

According to the 2001 census, the total aged (60 years and above) population of India is approximately 110 million, or approximately 11% of the total population. This percentage will be increased to 14.8% by the year 2020.[7] Many studies have had hinted that age is an important factor to which the CVD risk factors are related.[8] The fact remains that the problems of high blood pressure (BP) (essential hypertension) and obesity, considered to be the major risk factors for CVD, are increasingly assuming global importance.[9] Body weight is potentially an ideal modifiable risk factor in that weight tends to increase during the lifespan through late middle age. Age-related differences in the regional body composition are also documented by higher waist diameters,[10] higher waist-to-hip or waist-to-thigh ratios,[11] and lower girths in the limbs in older than in younger subjects.[12] Alterations in the lipid profile have also been associated with age. The TC, LDL-C, and atherogenic index were significantly higher and HDL-C was found to be lower in women older than 45 years when compared with those of women aged between 25 and 45 years.[13] Body fat distribution in addition, has physiologic and medical importance and may influence morbidity and mortality; therefore, it is important to assess the nutritional status or body composition of older people because of its role in ensuring a better quality of life and also its association with functional ability.[14‐16]

However, to the best of the authors' knowledge, virtually no study has been undertaken on people of Asian Indian origin to investigate the age and sex variation in the prevalence of CVD risk factors. Keeping this in mind, the present community-based cross-sectional study was aimed to investigate the age and sex variation in the prevalence of CVD risk factors in people of Asian Indian origin.

## MATERIALS AND METHODS

### Study population

The present community-based cross-sectional study was conducted between February 2007 and April 2010. A total of 682 (302 males and 380 females) participants aged 25–85 years took part in the study. A random sampling procedure using a local voters' registration list was followed to select the subjects. Prior to the actual commencement of the study, written information was communicated to select individuals and an appointment was requested at their respective houses. The subjects were categorized into 4 groups, namely, Group I (25–34 years; n = 141), Group II (35–44 years; n = 180), Group III (45–54 years; n = 171), and Group IV (55 years and above; n = 190). Women with prolonged chronic illness from diseases, such as polycystic ovarian syndrome, women on hormone therapy, as well as individuals with known illness, such as T2DM, CHD, were not included in the study. The Institutional Ethics Committee of the "Human Genetic Engineering Research Center" (HGERC), Kolkata, India, has approved the study. Written consent from the participants was also obtained prior to the actual commencement of the study.

### Anthropometric measures

Height, weight, and the circumferences of minimum waist (MWC) and maximum hip were collected using standard techniques.[17] Height and weight of lightly clothed subjects were measured to the nearest 0.1 cm and 0.1 kg, respectively. Circumferences were measured with an inelastic tape to the nearest 0.1 cm. Waist–hip ratio (WHR) was then calculated. The percentage of body fat (%BF) and body mass index (BMI) were measured using an Omron body fat analyzer (Omron Corporation, Tokyo, Japan). The validity of the analyzer was checked periodically by calculating BMI separately using the standard equation. It is noteworthy to mention that the Pearson's correlation coefficient (*r*) between the analyzer-operated BMI and the manually calculated BMI (weight in kg/height in m^2^ ) was 0.92 (r = 0.92; *P* < 0.0001).

### Blood pressure

Left arm systolic (SBP) and diastolic (DBP) blood pressure was taken from the participants with the help of an Omron MI digital electronic blood/pulse monitor (Omron Corporation, Tokyo, Japan). Two BP measurements were taken and averaged for analysis. A third measurement was only taken when the difference between the 2 measurements was ≥5 mmHg, and subsequently, the mean was calculated. A 5-minute relaxation period between measurements was maintained throughout the study. The working condition of the instrument was checked periodically using a mercury sphygmomanometer and a stethoscope.

### Metabolic profiles

A fasting blood sample (7 ml) was collected from 458 subjects for the determination of fasting blood glucose (FBG), TC, TG, and HDL. All subjects maintained an overnight fast of ≥12 h prior to blood collection. The plasma was separated within 2 h of blood collection using a microcentrifuge at 1000 rpm for about 20 min at room temperature. Estimation of FBG, TC, TG, and HDL were carried out using an ERBA Microscan ELISA Reader (Trans Asia Biomedicals Limited, Mumbai, India). TC:HDL and TG:HDL ratios were subsequently calculated. Low-density lipoprotein (LDL) and very low-density lipoprotein (VLDL) were then calculated by using the standard formula: LDL = TC ‒ (HDL + TG/5) and VLDL = TG/5. All the metabolic profiles were estimated in mg/dL (mg%) unit.

### Statistical analyses

Descriptive statistics, such as the mean and standard deviation (SD) were undertaken separately for each age group. Analysis of variance (ANOVA) was used to compare the 4 groups. Percentile distribution of BMI, WHR, TC, LDL, and TG:HDL ratio in the study population was also undertaken. Finally, Chi-square test was computed to compare the prevalence of high TG, high TC, high BP, and high BMI across the age groups.

All statistical analyses were performed using the SPSS (PC+ version 10). A *P* value of <0.05 (2-tailed) was considered as significant.

## RESULTS

Mean and standard deviation (SD) of anthropometric, obesity measures, lipids profiles, blood glucose, and BP in the study population are presented in Table 1. One-way ANOVA revealed significant differences for age, BMI, MWC, WHR, SBP, DBP, TC, TG, LDL, VLDL, and TC:HDL and TG:HDL ratios across the groups. But no significant group difference was observed for %BF, HDL, and FBG.

**Table 1:** Descriptive statistics of the study population (n = 682)

Variables	Group I (25–34 years)	Group II (35–44 years)	Group III (45–54 years)	Group IV (55+ years)
Age***	29.53 ± 03.0	39.58 ± 02.89	49.38 ± 02.91	62.72 ± 06.77
BMI***	23.43 ± 04.50	23.64 ± 04.01	22.93 ± 04.07	21.85 ± 04.06
MWC***	80.29 ± 12.66	84.50 ± 11.81	86.59 ± 10.01	87.21 ± 10.58
WHR***	00.85 ± 00.08	00.89 ± 00.09	00.93 ± 00.08	00.95 ± 00.07
% BF	28.47 ± 06.77	29.35 ± 09.27	29.86 ± 07.07	29.07 ± 07.15
SBP***	112.29 ± 14.35	122.14 ± 18.49	131.40 ± 20.97	141.63 ± 24.93
DBP***	74.53 ± 10.36	79.70 ± 10.78	83.253 ± 10.59	83.93 ± 11.36
TC***	186.85 ± 24.63	193.70 ± 29.44	198.59 ± 27.49	196.77 ± 25.00
TG**	134.29 ± 28.25	137.24 ± 25.74	143.75 ± 28.83	139.94 ± 26.13
HDL	46.17 ± 05.28	46.26 ± 06.09	45.13 ± 03.58	45.37 ± 04.81
LDL***	113.90 ± 22.18	119.92 ± 29.62	124.40 ± 28.46	123.39 ± 24.41
VLDL*	26.81 ± 05.63	27.45 ± 05.18	29.05 ± 06.80	28.04 ± 05.22
FBG	88.19 ± 08.89	89.86 ± 20.17	88.60 ± 18.24	93.77 ± 20.83
TC: HDL**	04.10 ± 0.74	04.29 ± 01.04	04.50 ± 01.04	04.40 ± 00.87
TG: HDL**	02.95 ± 0.749	03.05 ± 00.86	03.25 ± 00.90	03.14 ± 00.79

Anthropometry and blood pressure, Group I, n = 141; Group II, n = 180; Group III, n = 171; and Group IV, n = 190, Lipids and blood glucose, Group I, n = 94; Group II, n = 144; Group III, n = 155; and Group IV, n = 183, BMI, body mass index; MWC, minimum waist circumference; WHR, waist-hip ratio; % BF, percentage of body fat; SBP, systolic blood pressure; DBP, diastolic blood pressure; TC, total cholesterol; TG, triglyceride; HDL, high-density lipoprotein; LDL, low-density lipoprotein; VLDL, very low density lipoprotein; FBG, fasting blood glucose, Values are mean ± SD, Signifi cant group differences at

* *P* < 0.05

** *P* < 0.01

*** *P* < 0.001

Percentile distribution of obesity, central obesity measures, and lipids profiles according to age groups is presented in Table 2. The 25th percentile of BMI in Group I was 20.11 and Group IV was 18.80, whereas the 85th percentile of BMI in Group I and Group IV was 27.74 and 26.10, respectively. The 85th percentile of WHR in Group I and Group IV was 0.95 and 1.02, respectively. The 85th percentile of TC was 209.50 and 222.00 in Group I and Group IV, respectively. The 25th percentile of TG:HDL ratio in Group I and Group IV was 2.51 and 2.54, respectively.

**Table 2:** Percentiles distribution of cardiovascular disease risk factors

Variables Category	Percentiles	Group I	Group II	Group III	Group IV
BMI	25th	20.11	20.80	20.20	18.80
	50th	23.20	23.60	22.90	21.25
	75th	26.55	26.70	25.10	24.50
	85th	27.74	27.78	26.62	26.10
WHR	25th	0.79	0.82	0.87	0.90
	50th	0.86	0.91	0.93	0.95
	75th	0.92	0.97	0.95	1.00
	85th	0.95	1.00	1.01	1.02
TC	25th	170.75	176.00	184.50	182.00
	50th	188.00	190.00	195.00	195.50
	75th	198.50	201.00	210.00	208.00
	85th	20.9.50	221.50	225.00	222.00
LDL	25th	98.75	103.70	109.00	109.50
	50th	114.00	117.30	121.00	122.00
	75th	125.45	143.40	136.00	134.00
	85th	133.45	145.30	152.00	144.40
TG: HDL	25th	2.51	2.45	2.63	2.54
	50th	2.84	2.85	3.00	3.00
	75th	3.25	3.53	3.54	3.62
	85th	3.48	3.90	4.24	4.06

BMI, body mass index; BP, blood pressure; CVD, cardiovascular disease; SBP, systolic blood pressure; DBP, diastolic blood pressure; TC, total cholesterol; TG, triglyceride; FBG, fasting blood glucose

The prevalence of CVD risk factors according to age groups is presented in Table 3. It was observed that there were significant sex-specific group differences (male [χ^2^_ (12)_] = 29.22, *P* < 0.01 and female [χ^2^ _ (12)_] = 56.69, *P* < 0.001) for obesity, high BP, high TC, high TG, and high FBG. But no significant group-specific sex difference was evident for either of the risk factors, except for Group IV.

**Table 3:** Prevalence of cardiovascular disease risk factor by age groups and sex

Age groups	Sex	Prevalence of CVD risk factors+				
		Obesity	High BP	High TC	High TG	High FBG
Group I	Male	25	19	16	11	04
	Female	31	13	07	12	05
Group II	Male	39	37	25	21	09
	Female	35	39	27	23	15
Group III	Male	21	54	37	30	17
	Female	23	42	32	25	14
Group IV	Male	26	66	25	25	20
	Female	16	78	52	43	28

*BMI, body mass index; BP, blood pressure; CVD, cardiovascular disease; SBP, systolic blood pressure; DBP, diastolic blood pressure; TC, total cholesterol; TG, triglyceride; FBG, fasting blood glucose, +Obesity when BMI ? 25 kg/m2; high BP when SBP ? 130 or DBP ? 85 mmHg; high TC when TC ? 200 mg%; high TG when TG ? 150 mg%; high FBG when FBG ? 100 mg%, Sex-specifi c group difference for CVD risk factors: [?2 (12)] = 29.22; P < 0.01 (for male); [?2 (12)] = 56.69; P < 0.001 (for female), Group specifi c sex difference: Group I [?2 (4)] = 6.14, no signifi cant difference (P = 0.20); Group II [?2 (4)] = 1.70, no signifi cant difference (P = 0.80); Group III [?2 (4)] = 1.96, no signifi cant difference (P = 0.80); and Group IV [?2 (4)] = 15.03; P < 0.01*.

## DISCUSSION

It was observed in our study that there existed significant group differences for BMI, MWC, and WHR but no significant group difference for %BF was evident across the age groups. Despite having lower prevalence of obesity as defined by BMI, Asian Indians tend to have greater waist circumference and waist-to-hip ratios, thus having a greater degree of central obesity. Again, Asian Indians have more total abdominal and visceral fat for any given BMI, and for any given body fat they have increased insulin resistance.[18,19]

Change in BP is one of the commonly known physiologic changes in aging of man.[16] In our study, a significant group difference for SBP and DBP was also evident. Haarbo *et al*.[20] also observed high TC, LDL, VLDL, and TG levels with increasing age. In our study, we also found significant group differences for TC, LDL, VLDL, and TG:HDL ratio, but no difference was found for TG, HDL, FBG, and TC:HDL ratio across the age groups.

The prevalence of CVD risk factors, such as high BMI, high TC, high TG, and high BP as per age was also evident in our study suggesting that with age factors of metabolic syndrome, dyslipidemia is accumulated among them which in turn may lead them into the increases risk of CHD. And it is noteworthy to mention that CHD in Asian Indians occurs at least a decade earlier than that seen in Europeans or Americans.[21,22]

The results of the present investigation indicate that it is utmost essential to understand the underlying reasons involved in the causation of this significant trend of prevalence of cardiovascular risk as per age group so that national primary health care strategies for elderly people could be formulated better.

However, some shortcomings are associated with the present study, including small sample size; and therefore, are not representative of the Asian Indian population. Further studies are needed on other ethnic groups residing in rural as well as urban areas of India to determine whether a similar phenomenon exists among them. Moreover, investigation should be undertaken among the *Indian Diaspora* worldwide to elucidate if they also show age trends in CVD risk factors similar to sedentes in India or the local population of the respective countries.[22] Such studies would generate valuable information on the "nature–nurture" interaction involved in the aging process of CVD risk factors.
